# Nucleation Roadmap of Reduced Polyoxovanadate-Alkoxide Clusters

**DOI:** 10.1021/acs.inorgchem.4c04759

**Published:** 2025-02-20

**Authors:** S. Genevieve Duggan, S. M. Gulam Rabbani, Pere Miró

**Affiliations:** † Department of Chemistry, University of Iowa, Iowa City, Iowa 52242, United States; ‡ Department of Chemistry, University of South Dakota, Vermillion, South Dakota 57069, United States

## Abstract

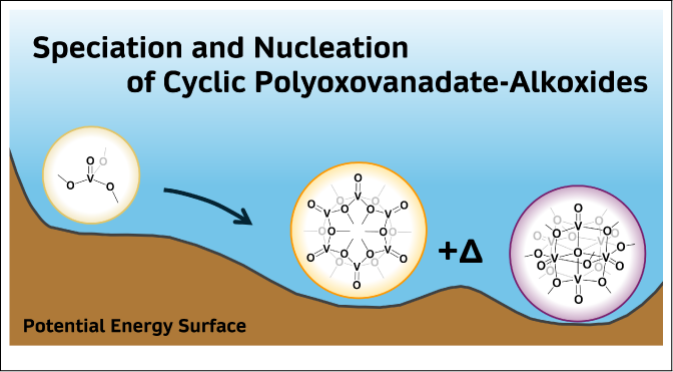

Polyoxovanadate-alkoxides are a growing family of earth-abundant first-row transition metal polynuclear species highly promising for their tunable redox properties. The speciation and nucleation chemical space of these species is divided into two groups: 1) fully oxidized V(V) monomeric precursors that aggregate into Lindqvist-type clusters and 2) reduced V(IV) precursors forming cyclic structures. The nucleation of cyclic polyoxovanadate-alkoxides with varying alkyl chain lengths, the impact of the presence of templating anions, and their subsequent evolution to the Lindqvist-type congener were studied by using density functional theory. The evolution of cyclic polyoxovanadate-alkoxides to oxygen-vacant cluster structures was found to be endergonic, in agreement with previous experimental work. Moreover, the reactivity with traces of water in alcohol solvents was confirmed to be the main thermodynamic driving force toward the formation of the mixed-valent Lindqvist-type polyoxovanadate species.

## Abstract 2

The nucleation of cyclic polyoxovanadate-alkoxides with varying alkyl chain lengths, the impact of the presence of templating anions, and their subsequent evolution to the Lindqvist-type congener were studied by using density functional theory.

## Introduction

Polyoxometalates (POMs) are a diverse class of inorganic compounds comprised of metal centers linked and terminated by oxo ligands.^1,2^ Elements from groups V to VI are the most common metals present in POMs; however, almost all elements in the periodic table have been incorporated into their structures.^3^ POMs have a wide range of energy-related applications due to their high stability, chemical diversity, functionalization, and tunable physicochemical properties (e.g., catalytic and electrochemical properties).^4,5^ In the POM chemical space, molybdenum- and tungsten-based POMs are by far the most common, but there are a significantly smaller number of species with vanadium as their main metal center, known as polyoxovanadates (POVs).^6−12^ There is special interest in POVs because of vanadium’s abundance in earth’s crust, conformational stability, and redox profile. In aqueous solution, the dominant high-nuclearity iso-polyoxovanadate is ubiquitous decavanadate ([V_10_O_26_]^6–^), while the Lindqvist structure, which is common among tungsten and molybdenum POMs, has yet to be isolated experimentally.^13^

**Figure 1:**
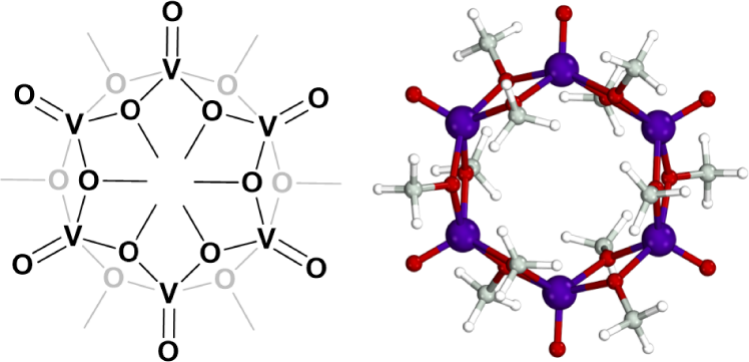
Schematic representation (left) and ball-and-stick representation (right) of the hexameric [(V(O))_6_(μ_2_-O–CH_3_)_12_] cyclic polyoxovanadate-alkoxide cluster with ethoxide ligands. Color code: vanadium in purple, oxygen in red, carbon in gray, and hydrogen in white.

An approach to move beyond the chemical space dominated by decavanadate in aqueous media is to use alternative green solvents such as alcohols. Under these conditions, the formation of bridging μ_2_-O moieties is quenched in favor of bridging alkoxides μ_2_-O(CH_3_). Furthermore, the overall charge of POV-alkoxides is reduced with respect to traditional polyoxovanadates due to the lower charge of alkoxide ligands compared to that of oxo ones. For example, mixed-valent hexanuclear polyoxovanadate (POV) alkoxide clusters [(O_6_)(O)(O–CH_3_)_12_]^(4–*n*)+^ have emerged as stable Lindqvist hexavanadate alternatives.^14−21^ However, under certain experimental conditions, a family of reduced cyclic POV-alkoxide clusters [(V(O))_6_(μ_2_-OR)_12_] (R = −CH_3_, −C_2_H_5_) has been isolated instead. These cyclic structures have the same number of bridging alkoxide ligands as the Lindqvist POV alkoxides but one fewer oxo ligand (Figure 1).^22^ These species are not the thermodynamic minima in the POV-alkoxide chemical space since they ultimately evolve into oxygen-deficient [(V^IV^O)_6_(μ_2_-O–CH_3_)_12_] clusters and finally into Lindqvist-type POV-alkoxide species.^23^

These cyclic POV-alkoxides are structurally and electronically different from prior Lindqvist alkoxides. They resemble a cyclic hexameric structure and contain six V(IV) centers, while the Lindqvist-type alkoxides are mixed-valent. The vanadium V(V)/V(IV) redox couple appears to be key in driving and controlling the nucleation of POV-alkoxide species, with the presence of V(V) monomeric precursors in solution favoring the aggregation into mixed-valent Lindqvist-type clusters, while the presence of fully reduced V(IV) precursors in solution tends to lead to the formation of fully reduced cyclic structures.

With respect to judiciously tuning POV-alkoxides for selected applications, a further study into their nucleation is required, since our knowledge regarding their assembly is sparse. Mechanistic studies of POV-alkoxides speciation and nucleation are challenging due to the complex speciation, myriad of nucleation pathways, and spontaneous self-assembly, which are all ubiquitous in POM synthesis.^24−31^ Templating groups have been successfully employed in POM synthesis to generate predictable structures in solution, but the use of a directing or templating agent is rare in POV-alkoxide synthesis. Most of the Lindqvist-type assemblies of POV-alkoxides have been synthesized serendipitously without any templating or directing agent at relatively high temperatures. Interestingly, cyclic POV-alkoxides can be generated at relatively low temperatures in the presence of halide anions, which in the case of cyclic POV-methoxide species are incorporated into their crystal structures at the center of the ring. This indicates that halide ions could act as templating agents during the growth of cyclic POV-alkoxides. However, those with ethoxide bridging ligands lack the central chloride agent found in the methoxide counterpart. Therefore, it is critical to understand whether anions play a role in the nucleation of cyclic POV-alkoxides. If chloride dictates the formation of hexameric cyclic POV-alkoxides, then another halogenic group might also direct the formation of different cyclic POV-alkoxides. If chloride anions do not act in this way, other strategies to control cluster growth must be pursued. Experimentally, it has been determined that the nucleation of cyclic POV-alkoxides is sensitive to increases in the alkoxide chain length. Therefore, larger alkoxide chains could result in the formation of smaller cyclic species.^32^

Herein, we investigate the nucleation thermodynamics of fully reduced cyclic POV-alkoxides. Specifically, we explored the impact of alkoxide chain length and the extent to which halide anions act as templating agents. Furthermore, we also investigated the electronic structure and evolution of the cyclic POV-alkoxides, first into the oxygen-deficient cluster isomer and then into the mixed-valent Lindqvist-type thermodynamic product, [(V(O))_6_((μ_6_-O))(μ_2_-OCH_3_)_12_].

## Computational Details

Density functional theory (DFT) calculations were performed using the Turbomole package with the PBE0 exchange-correlation functional in conjunction with the multipole accelerated resolution of the identity approximation, while employing an m4 grid.^33−37^ The def2-TZVP basis set was used for all atoms.^38^ All species with unpaired electrons were considered to be in their high spin state. Dispersion effects were included using Grimme’s D3 correction.^39^ The nature of all stationary points and transition states was verified by analytic computation of vibrational frequencies. A Truhlar-type quasi-harmonic correction was used for all frequencies below 100 cm^–1^ in which they are shifted to 100 cm^–1^.^40^ Molecular partition functions were used in the computation of 298.15 K thermal contributions to Gibbs free energy, employing the standard ideal-gas, free-rotor, and quasi-harmonic oscillator approximations. A vibrational scaling factor of 0.982 was used for all frequencies. Standard state conditions were included for all of the species. All calculations were performed without symmetry constraints. Solvent effects were included in PBE0-D3/def2-TZVP single-point calculations using the COSMO solvation model with tetrahydrofuran parameters on gas-phase optimized geometries obtained at the PBE0/def2-TZVP level of theory.^41^ Single-point calculations were also performed with the B3LYP exchange-correlation functional in tetrahydrofuran to assess functional sensitivity.^42−44^

## Results and Discussion

### Initial Nucleation

The Lindqvist-like [(V(O))_6_(μ_6_-O)(μ_2_-O–CH_3_)_12_] species were obtained via solvothermal synthesis starting from monomeric V(V) precursors in the presence of mild reducing agents.^14−19^ On the contrary, the synthesis of cyclic polyoxovanadate species involves a similar V(V) precursor and a stronger reducing agent. Experimentally, the addition of solid [Bu_4_N][BH_4_] turned the solution to a dark green color, indicative of the reduction of the monomeric precursor to V(IV).^22,23^ Therefore, instead of beginning our nucleation pathway from the monomeric [V^V^(O)(O–CH_3_)_3_] species present in the nucleation of the Lindqvist-type POV-alkoxide, the cyclic species were modeled by starting with a [V^IV^(O)(O–CH_3_)_2_(CH_3_OH)] precursor consistent with the observed V(IV) oxidation state.

**Figure 2:**
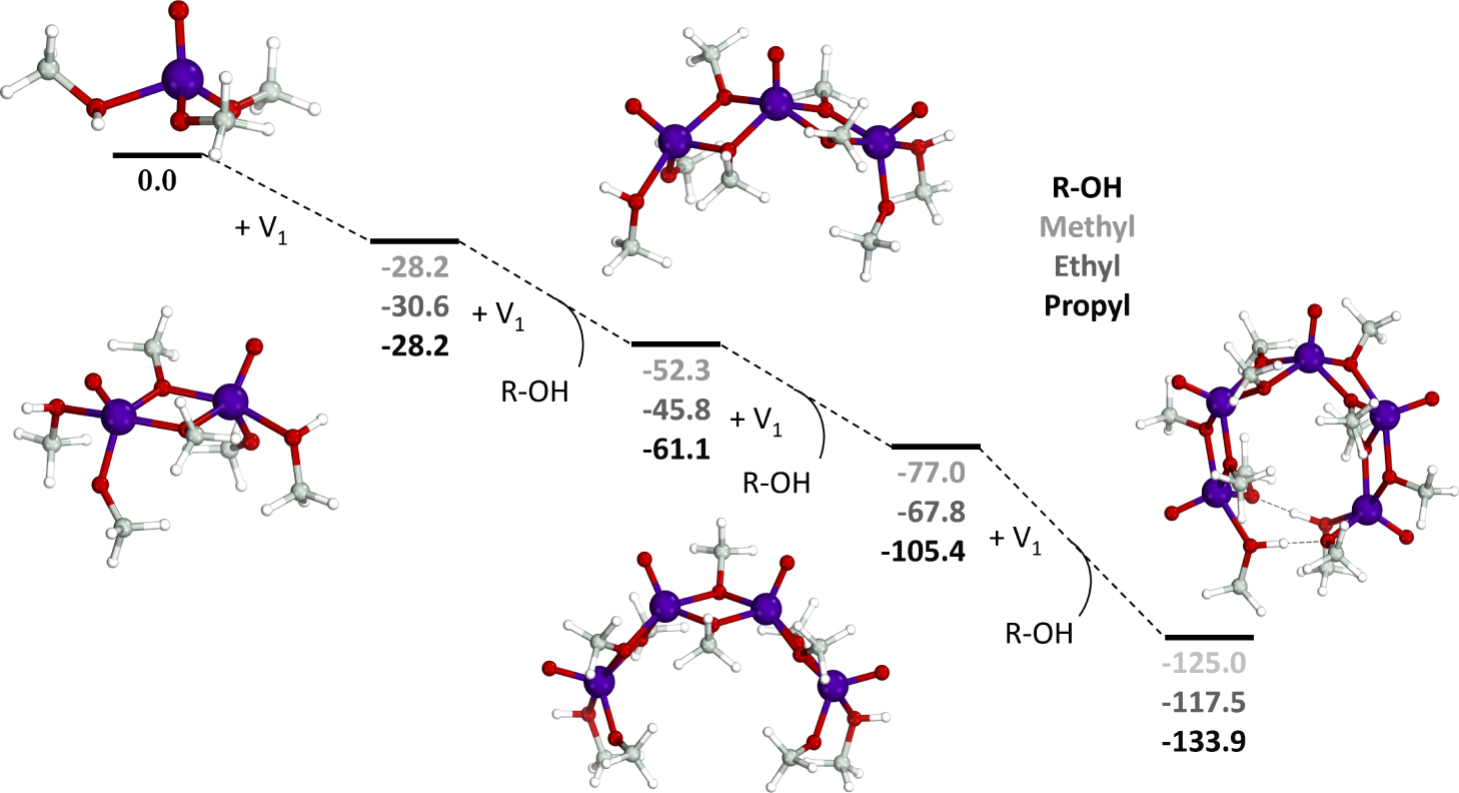
Stepwise nucleation of the monomeric [(V^IV^O)(O–R)_2_(R–OH)] species to the pentameric [(V^IV^O)_5_(μ_2_-O–R)_8_(O–R)_2_(R–OH)_2_] species with different alkoxide chain lengths (R = −CH_3_, −CH_2_–CH_3_, or −(CH_2_)_2_–CH_3_). Gibbs free energies with respect to free monomers reported in kcal/mol. Color code: vanadium in purple, oxygen in red, carbon in gray, and hydrogen in white.

The first step in the nucleation of POV-alkoxides is a dimerization reaction with a small entropic barrier of 4 kcal/mol (Figure S1). Each subsequent increase in nuclearity involves the addition of a monomeric precursor and the release of a methanol ligand. Figure 2 shows the Gibbs free energy reaction pathway from the monomeric [(V^IV^O)(O–CH_3_)_2_(CH_3_OH)] precursor to the pentameric [(V^IV^O)_5_(μ_2_-O–CH_3_)_8_(O–CH_3_)_2_(CH_3_OH)_2_] species. In marked contrast to the initial nucleation of the mixed-valent Lindqvist POV-akoxide where multiple endergonic reactions must take place to increase the species’ nuclearity, each reaction step in the reduced cyclic POV-alkoxide is exergonic.^24^ This confirms the initial experimental hypothesis that the reduction of V(V) to V(IV) enhances the formation of a larger nuclearity species. As expected, the increase in the alkyl chain increases the thermodynamic stability of larger nuclearity species, which can be explained by the larger dispersion interactions in longer chains. However, steric effects are significantly different in the methyl case, and thus, there is the lack of a clear trend in the nucleation energetics going from methyl to propyl species.

### Ring-Closing Step

**Figure 3:**
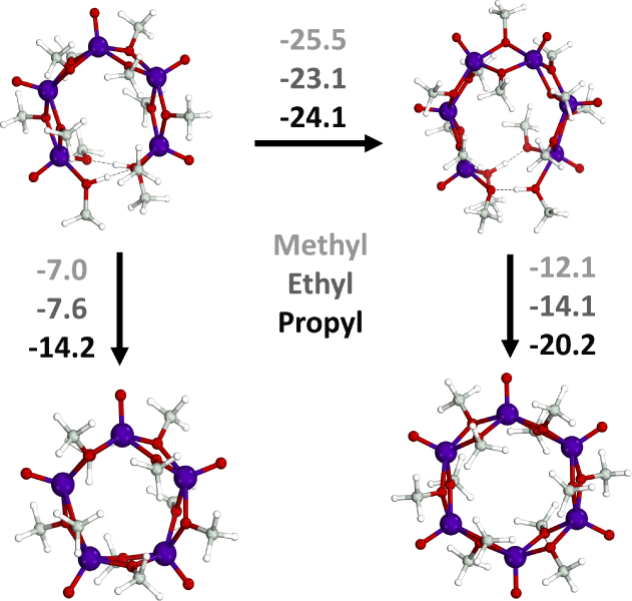
Nucleation of pentameric [(V^IV^O)_5_(μ_2_-O–CH_3_)_8_(O–CH_3_)_2_(CH_3_OH)_2_] POV-methoxide species into the pentameric or hexameric cyclic species. Gibbs free energies are in kcal/mol. Color code: vanadium in purple, oxygen in red, carbon in gray, and hydrogen in white.

The formation of a closed cyclic structure was considered via two possible pathways: the formation of a cyclic pentamer or an increase in nuclearity to form a cyclic hexamer, as shown in Figure 3. The ring-closing mechanism proceeds through the release of two alcohols to close the structure. Therefore, this is favored entropically due to the alcohol decoordination but disfavored enthalpically due to ring strain. The open-chain pentamer has significant curvature, with the terminal ends of the structure held in place by two hydrogen bonds between the −OH of the alcohol and the oxygen on the neighboring alkoxide. The ring-closing step was found to be more thermodynamically favorable in the hexameric species than the pentameric species, specifically by 3.1 kcal/mol in the case of the POV-methoxide. Increasing the alkyl chain length from the methoxide bridging ligands to ethoxide and propoxide bridging ligands had a stabilizing effect on the formation of pentameric species. The trend in the Gibbs free energy in the pentameric ring-closing reaction may include contributions from two driving forces. The increasing bulk of the alkoxide ligands imparts more curvature in the open-chain structure, making smaller cyclic species more accessible. The longer alkyl chains also stabilize the formation of larger species, such as the hexameric species, through the increase of the dispersion interactions between the nucleating intermediates.

### Effect of Templating Anions

The presence of a chloride in the [(V^IV^O)_6_(μ_2_-O–CH_3_)_12_] X-ray crystal structure opened up the possibility of the halide having a templating effect during the growth of species or the thermodynamic stabilization of the nucleation intermediates or final product. The influence of templating halide anions such as fluoride and chloride was considered, beginning with the open chain pentamer. The ion association Gibbs free energies of F^–^ and Cl^–^ to the bare pentameric [(V^IV^O)_5_(μ_2_-O–CH_3_)_8_(O–CH_3_)_2_(CH_3_OH)_2_] species are −41.4 and −19.9 kcal/mol, respectively. This suggests that the use of a small halide anion such as fluoride would lead to the formation of stronger ion pairs with the POV-alkoxide species. In the presence of paired halide anions, the formation of hexameric species was still found to be the thermodynamic minimum; however, the fluoride templating anion had a significant stabilizing effect on the formation of cyclic pentamers. The chloride ion, by contrast, resulted in an endergonic reaction for the pentameric ring-closing mechanism. The use of the chloride ion as a templating agent becomes increasingly exergonic with extended alkyl chain lengths, which is in agreement with an increase in the dispersion interaction of the alkyl chains. The central chloride ion has only been experimentally observed in the crystal structure of the POV-methoxide species; it is absent in X-ray crystal structures with longer alkyl chains. We attribute this to the bulkiness of longer alkyl chains, which make the incorporation of such an ion harder. This trend could be subdued using fluoride, given its smaller ionic radius and higher ion association free energy with POV-alkoxide species (Table 1).

**Table 1:** Reaction Energy for the Formation of Pentameric [(V^IV^O)_5_(μ_2_-O–CH_3_)_10_] (V_5,cyclic_) and Hexameric [(V^IV^O)_6_(μ_2_-O–CH_3_)_12_] (V_6,cyclic_) Cyclic Species and the Nucleation from the Open Pentameric [(V^IV^O)_5_(μ_2_-O–CH_3_)_8_(O–CH_3_)_2_(CH_3_OH)_2_] (V_5_) to the Open Hexameric [(V^IV^O)_6_(μ_2_-O–CH_3_)_10_(O–CH_3_)_2_(CH_3_OH)_2_] Species with Different Alkyl Chains (R = −CH_3_, −CH_2_–CH_3_, or −(CH_2_)_2_–CH_3_)^a^

	V_5_ → V_5,cyclic_			Nucleation V_5_ to V_6_			V_6_ → V_6,cyclic_		
Alkyl Chain	None	F^–^	Cl^–^	None	F^–^	Cl^–^	None	F^–^	Cl^–^
–CH_3_	–7.0	–18.1	+11.1	–25.5	–23.0	–25.7	–12.1	–17.2	–18.2
–CH_2_–CH_3_	–7.6	–18.1	+7.1	–23.1	–35.9	–28.2	–14.1	–7.2	–17.1
–(CH_2_)_2_–CH_3_	–14.2	–33.4	–2.1	–24.1	–41.5	–40.0	–20.2	–16.0	–19.1

a Reaction energies are included both with and without halide anions. Gibbs free energies in kcal/mol.

### Conversion of Cyclic to Lindqvist-Type Polyoxovanadate-Alkoxide Species

**Figure 4:**
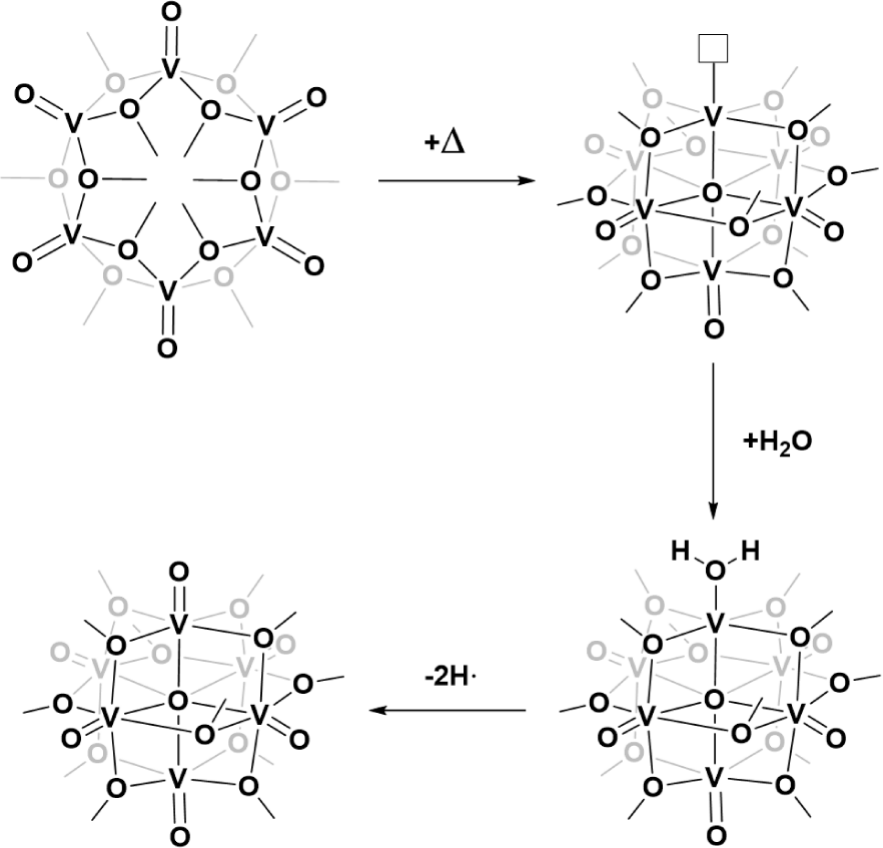
Proposed mechanism for the formation of Lindqvist-type [(V^IV^O)_4_(V^V^O)_2_(μ_6_-O)(μ_2_-O–CH_3_)_12_] species from the cyclic [(V^IV^O)_6_(μ_2_-O–CH_3_)_12_] species. The oxo-vacancy is highlighted with a square box.

Meyer et al. demonstrated that the cyclic [(V^IV^O)_6_(μ_2_-O–CH_2_CH_3_)_12_] species can be thermally transformed into the Lindqvist-type [(V^IV^O)_4_(V^V^O)_2_(μ_6_-O)(μ_2_-O–CH_2_CH_3_)_12_] species (Figure 4).^23^ Therefore, we investigated the formation of Lindqvist-type species from hexameric cyclic species. The cyclic species have been identified as the kinetic product toward the formation of oxygen-deficient [(VO)_6_(μ_2_-O–CH_2_CH_3_)_12_] Lindqvist-type clusters. The conversion of the cyclic POV-methoxide species to the oxygen-deficient Lindqvist-type structure was endothermic and endergonic by +15.2 and +16.2 kcal/mol, respectively. This indicates a small entropic contribution to the energetics associated with this transformation; however, the mechanism for such a structural rearrangement remains unknown and will be the focus of future studies. The endoenergetic character of this transformation is in agreement with the experimental procedure occurring at 125°C. Furthermore, there is a significant change in the electronic structure of the oxygen-deficient Lindqvist-type cluster, in terms of the oxidation state of the vanadium centers. The spin density of the oxygen-deficient Lindqvist-type cluster confirms mixed-valent character at the vanadium centers with one V(III), one V(V), and four V(IV) centers (Figure S2). This is due to an intramolecular redox process that takes place, leading to the reduction of the vanadium center with the oxygen vacancy and the oxidation of the vanadium center trans to it.

The reaction of the oxygen-deficient Lindqvist-type cluster with traces of water present in the alcohol solvent to form [(V^III^(H_2_O))_5_(V^IV^O)_4_(V^V^O)(μ_6_-O)(μ_2_-O–CH_3_)_12_] is exergonic by −6.5 kcal/mol. The generation of hydrogen from the electrolysis of water has been proposed as a green and sustainable hydrogen fuel source when coupled with a renewable energy source.^45^ In this instance, the electron-rich POV-alkoxide cluster could be the electron source for water reduction. This would lead to the formation of the mixed-valent [(V^IV^O)_4_(V^V^O)_2_(μ_6_-O)(μ_2_-O–CH_3_)_12_] and the production of two hydrogen-atom equivalents.^24^ The average bond dissociation Gibbs free energy (BDFE) of the vanadium-water O–H bonds is 58.9 kcal/mol, which closely resembles experimental values previously reported for reduced polyoxovanadate-alkoxide clusters.^46,47^ However, these values remain too high to spontaneously form molecular hydrogen.^48^ Nevertheless, these species are highly promising as sources of reactive hydrogen-atom equivalents at molecular metal oxide surfaces from “green” substrates (e.g., H_2_O).

## Conclusions

Herein, we have explored the nucleation pathway of reduced POV-alkoxide species, taking into consideration the alkoxide chain length and the effect of templating agents. Starting with reduced V(IV) monomeric precursors, a series of consecutive exergonic reactions proceed up to the formation of the open-chain pentamer. The cyclic hexameric POV-alkoxide is the thermodynamically most stable product, regardless of the alkyl chain length or halide ion. We observed that longer alkyl chains and smaller halide anions stabilize smaller rings via dispersion and electrostatic interactions; however, in all the cases, such stabilization is not enough to stabilize pentameric structures over hexameric ones. The evolution of cyclic polyoxovanadate-alkoxides to oxygen-vacant cluster structures was found to be endergonic, in agreement with previous experimental work. Moreover, the reactivity with traces of water in alcohol solvents was confirmed to be the main thermodynamic driving force toward the formation of the mixed-valent Lindqvist-type polyoxovanadate species. This work completes the nucleation of hexavalent POV-alkoxide, which we aim to leverage in the future to create thermodynamic and kinetic speciation and nucleation models involving a single chemical space with both V(V) and V(IV) species.

## Supporting Information Available

Intrinsic reaction coordinate, spin density, dispersion-free energetics, and structures (PDF)

## Dedication

This work is dedicated to the memory of Prof. Achim Müller.
