# Supplementary material for: Nucleation Roadmap of Reduced Polyoxovanadate-Alkoxide Clusters
Source: Inorg Chem. 2025 Feb 20;64(8):3790–5. doi: 10.1021/acs.inorgchem.4c04759 (PMC11881037; doi:10.1021/acs.inorgchem.4c04759)
Supplement: Supplementary file 1 — ic4c04759_si_001.pdf [file ic4c04759_si_001.pdf]

Nucleation Roadmap of Reduced Polyoxovanadate-Alkoxide Clusters

S. Genevieve Duggan,[†,‡] S. M. Gulam Rabbani,[‡] and Pere Miró[*,†,‡]

†*Department of Chemistry, University of Iowa, 52242 Iowa City, IA, USA*

‡*Department of Chemistry, University of South Dakota, 57069 Vermillion, SD, USA*

E-mail: pere-miro@uiowa.edu,pere.miro@usd.edu

All calculations are available in a ioChem-BD repository.

https://iochem-bd.bsc.es/browse/handle/100/330306

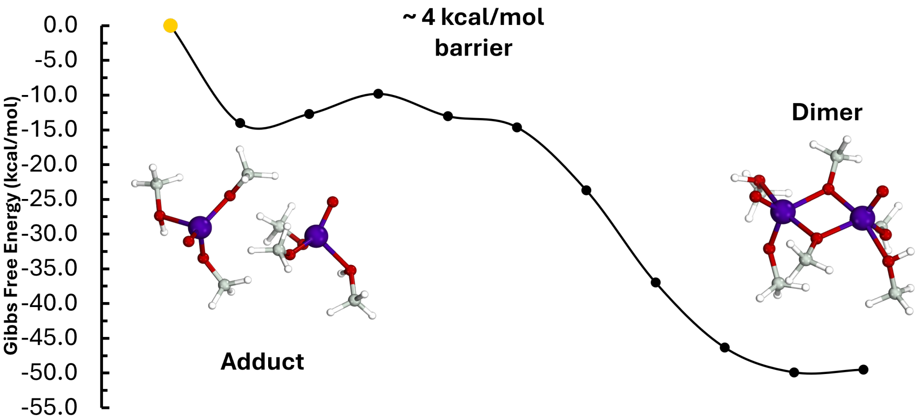

Figure S 1: Intrinsic reaction coordinate of the nucleation of monomeric $[(V^{IV}O)(O\text{-}R)_2(R\text{-}OH)]$ species (yellow dot) to the dimeric $[(V^{IV}O)_2(O\text{-}R)_4(R\text{-}OH)_2]$ species with R=-CH_3.

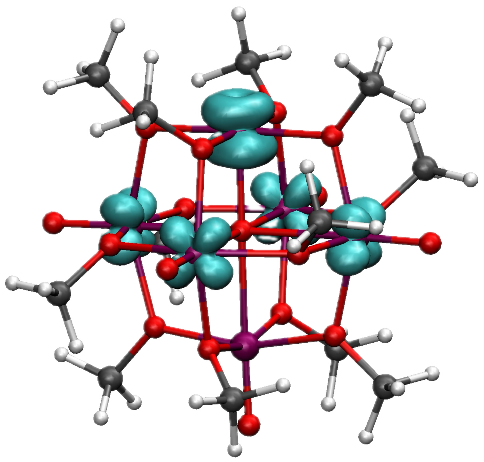

Figure S 2: Spin density plot of oxygen deficient $[(VO)_6(\mu_2\text{-O-CH}_3)_{12}]$ species at RI-PBE0/def2-TZVP level of theory.

Table S 1: Difference in Gibbs free energy of polyoxovanadate-alkoxide species with and without the Grimme D3 dispersion corrections in kcal/mol.

**-R**	$\mathbf{V}_2$	$\mathbf{V}_3$	$\mathbf{V}_4$	$\mathbf{V}_5$
$-CH_3$	-20.0	-28.9	-39.3	-59.4
$-CH_2CH_3$	-31.9	-49.4	-66.8	-87.8
$-CH_2CH_2CH_3$	-43.8	-66.9	-94.8	-116.3

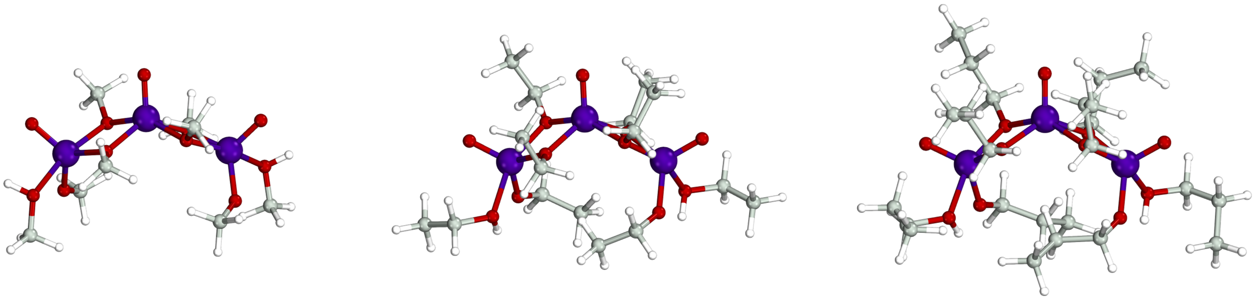

Figure S 3: Trimeric polyoxovanadate-alkoxides with methoxide (left), ethoxide (center), and propoxide (right) bridging ligands.

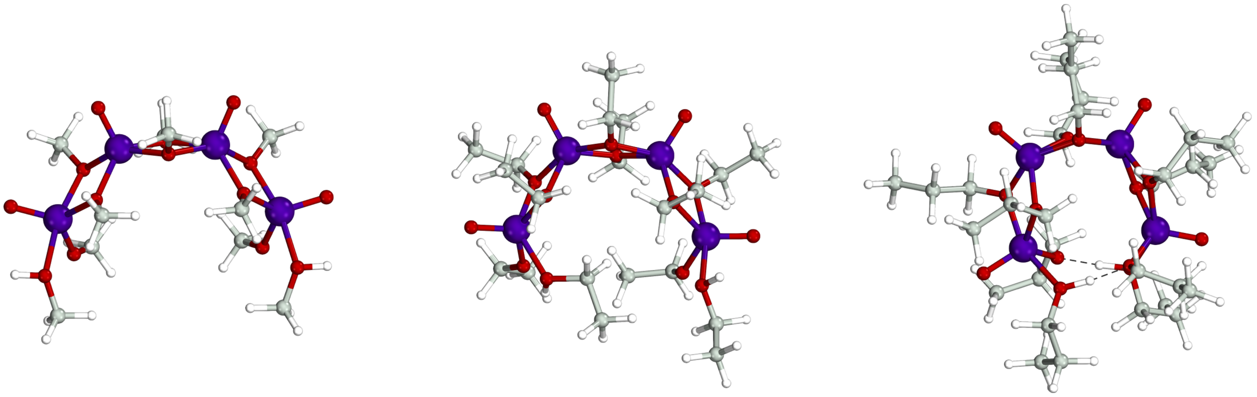

Figure S 4: Tetrameric polyoxovanadate-alkoxides with methoxide (left), ethoxide (center), and propoxide (right) bridging ligands.

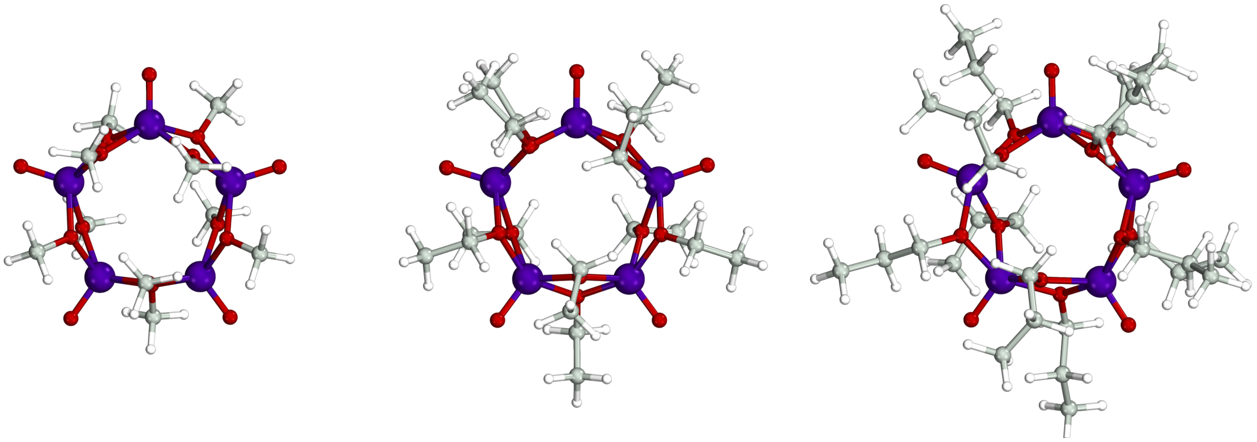

Figure S 5: Pentameric polyoxovanadate-alkoxides with methoxide (left), ethoxide (center), and propoxide (right) bridging ligands.

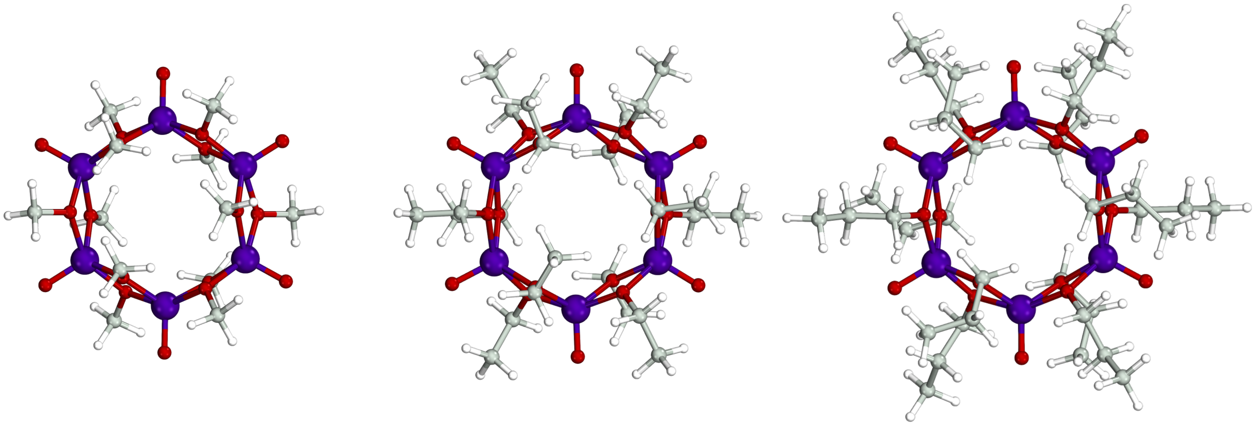

Figure S 6: Hexameric polyoxovanadate-alkoxides with methoxide (left), ethoxide (center), and propoxide (right) bridging ligands.
